# Assessment of Risk Factors and the Relationship between Hypothyroidism with Hypertension in Diabetes Mellitus Patients: A Cross-Sectional Community-Based Study

**DOI:** 10.3390/jpm13081231

**Published:** 2023-08-03

**Authors:** Mohammad Abu Shaphe, Mohammed M. Alshehri, Bushra Alfaifi, Mohammed A Aljahni, Vandana Esht, Shazia Malik, Marissa J Bautista, Abdulfattah S. Alqahtani, Ausaf Ahmad, Ashfaque Khan, Aafreen Aafreen, Abdur Raheem Khan

**Affiliations:** 1 Department of Physical Therapy, College of Applied Medical Sciences, Jazan University, Jazan 45142, Saudi Arabia; mshaphe@jazanu.edu.sa (M.A.S.); moalshehri@jazanu.edu.sa (M.M.A.); balfaifi@jazanu.edu.sa (B.A.); vandanaesht@jazanu.edu.sa (V.E.); samalik@jazanu.edu.sa (S.M.); mbautista@jazanu.edu.sa (M.J.B.); 2 Physical Education Department, Jazan University, Jazan 45142, Saudi Arabia; maljahni@jazanu.edu.sa; 3 Department of Rehabilitation Sciences, College of Applied Medical Sciences, King Saud University, Riyadh 11451, Saudi Arabia; abalqahtani@ksu.edu.sa; 4 Department of Community Medicine, IIMS&R, Integral University, Lucknow 226026, India; ausafahmad86@gmail.com; 5 Department of Physiotherapy, Integral University, Lucknow 226026, India; physiorehan@gmail.com (A.K.); aafreen@iul.ac.in (A.A.)

**Keywords:** hypothyroidism, hypertension, diabetes mellitus

## Abstract

(1) Background: hypertension (HTN) and diabetes mellitus (DM) represent two widely noncommunicable diseases that are prevalent globally, and they often correlate with chronic health issues. There has been an acknowledged connection between diabetes, hypertension, and hypothyroidism for quite some time. However, the extent of thyroid dysfunction among the diabetic population is not uniform and significantly differs across different research studies. This study was conducted with the objective of identifying the risk factors associated with hypothyroidism as well as assessing the relationship between hypothyroidism and hypertension in patients with diabetes. (2) Materials and Methods: Participants aged 18 years and above were included in this study, while pregnant women were excluded. Trained health professionals measured sociodemographic, behavioural, food practices, and anthropometric information about the participants. Each respondent sought medical advice regarding their health, and a face-to-face interview enabled them to express concern about the likelihood of being diagnosed with diabetes mellitus and hypertension. (3) Results: The study encompassed 640 participants, with an average age of 49.20 ± 13.0 years. Among these participants, 65.5% were female, and 34.5% were male. Of the total, 31.25% were diagnosed with diabetes mellitus, and 18.75% had hypertension. Interestingly, co-occurrence of both conditions was observed in 9.68% of the population. A comparison of thyroid function and indicators of blood sugar levels yielded consistent results across the different patient groups. Specifically, for diabetes mellitus (DM) patients, the average levels were 3.4 ± 9.8 pg/mL for fT3, 0.9 ± 0.7 ng/dL for fT4, 3.3 ± 6.2 μiU/mL for TSH, 153.1 ± 68.0 mg/dL for fasting plasma glucose (FPG), 213.2 ± 97.2 mg/dL for postprandial glucose (PPG), and 8.3 ± 3.2% for HbA1c. (4) Conclusion: It is concluded that patients with hypertension had a significant prevalence of diabetes mellitus. Subclinical hypothyroid subjects must be frequently screened for hypertension. Of 120 individuals with hypertension, 45 (37.5%) were also diagnosed with diabetes. This co-occurrence was significantly higher in subjects aged over 50 years (26.7%), in the lower socio-economic class (18.5%), and among those who were married (14.7%). Additionally, patients with hypertension exhibited a high prevalence of diabetes across different educational backgrounds and occupations, with the highest prevalence among postgraduates (37.5%) and professionals (24.0%), respectively. These findings highlight the need for an integrated approach to the management of hypertension and diabetes, particularly in high-risk demographics.

## 1. Introduction

As the Asian population continues to live longer, the focus of public health has pivoted towards non-communicable diseases (NCDs) [1]. The prevalence of hypertension is growing worldwide, attributed to the ageing population and exposure to lifestyle risk factors, such as unhealthy eating habits. However, the rise in hypertension is not globally uniform [2]. In the case of diabetes mellitus (DM) and hypertension (HTN), two prominent NCDs contribute substantially to morbidity and healthcare costs. NCDs, often dubbed the “invisible epidemic”, account for more deaths than maternal, perinatal, and infectious diseases. Projections for 2025 estimate global hypertension cases will increase to 1.5 billion, with Sub-Saharan Africa accounting for 125.5 million [1]. Patients diagnosed with diabetes and HTN frequently report symptoms of anxiety and depression [3,4]. Depression impacts between 10 and 30% of individuals with diabetes globally, resulting in impaired glucose control, reduced medication adherence, elevated cardiovascular morbidity, and increased mortality [5]. The investigation of the relationship between hypothyroidism and HT or DM was primarily triggered by the observation that patients with hypertension often exhibit thyroid dysfunction, particularly subclinical hypothyroidism. This thyroid dysfunction can cause various metabolic abnormalities and contribute to the development and progression of hypertension. Moreover, it has been found that suboptimal management of thyroid conditions can lead to uncontrolled blood pressure and pose additional health risks. Additionally, anxiety exacerbates complications of the micro and macrovascular systems in diabetic patients [6]. Hypertension has a recognised association with anxiety; however, its relationship with depression is more complex. While some studies show no connection between HTN and depression [4], others point to a significant link [7]. Patients with hypertension often exhibit thyroid dysfunction, specifically subclinical hypothyroidism, which may exacerbate their condition. This thyroid dysfunction can cause various metabolic abnormalities and contribute to the development and progression of hypertension. Consequently, suboptimal management of thyroid conditions can lead to uncontrolled blood pressure and pose additional health risks.

Assessment of thyroid functioning has been substantially improved by increased TSH sensitivity and specificity. HTN risk may be increased by thyroid disease, including both hypothyroidism and hyperthyroidism. It is debatable whether subclinical hypothyroidism, a minor form of thyroid malfunction, affects blood pressure [8]. While existing research indicates a complex interrelationship between hypothyroidism, hypertension, and diabetes mellitus, there is a noted discrepancy in the prevalence of thyroid dysfunction among the diabetic population across various studies. Thus, this study aims to bridge this knowledge gap by specifically identifying the risk factors associated with hypothyroidism and discerning the relationship between hypothyroidism and hypertension in patients with diabetes mellitus within a community setting. This will contribute to a clearer understanding of the comorbidity of these conditions, ultimately informing better management and intervention strategies for affected populations.

## 2. Materials and Methods

The methodological approach for this research was defined as cross-sectional, descriptive, community-focused research [1]. Adults aged 18 years and older, of both sexes, with or without diabetes mellitus (DM) from chosen localities, were incorporated into the sample [2]. Regarding patient eligibility, our main inclusion criteria were age (18 years and older) and residing within the selected localities, irrespective of their DM or hypertension (HT) status. Individuals younger than 18 years, pregnant women, those suffering from cognitive impairments, and severely sick patients were excluded [3]. We acknowledged that the prevalence of HT and DM increases with age, and our inclusive age threshold aimed to account for this fact. At the same time, our broad selection from the community was intended to reflect a true cross-section of the population, including those with increased risk due to age. The investigation was conducted within both impoverished and non-impoverished areas of the town [4]. Estimating a probable prevalence of hypertension at 50%, the study allowed an error margin of 5% and ensured a 95% confidence level [5]. A minimum sample size of 384 was determined by the computations [5]. This figure was increased by 1.5 times considering the design effect [6]. Based on an anticipated non-response rate of 10%, 640 respondents were selected for the study [7].

The research was conducted in the jurisdiction of a teaching hospital’s Urban Health Training Centre (UHTC), which catered to both deprived and non-deprived areas [8]. Locations were chosen through simple random sampling to achieve the desired sample size [9]. Prior to participating in the study, all individuals provided written consent. However, certain groups were excluded from the study due to specific criteria. These exclusion criteria included individuals diagnosed with cancer within the last five years, individuals currently experiencing fever, viral infections, or neck pain, and those taking medications that could potentially influence thyroid functions [10,11]. Approval for the study was granted by the University’s Research Ethics Committee (IEC/IIMS&R/2021/25).

The investigators conducted a door-to-door survey, explaining the study’s purpose to prospective participants and seeking verbal consent before gathering demographic, behavioural, dietary, and anthropometric data using pre-tested interview schedules [12]. Every eligible individual in the selected households was included in the study [13]. The individuals involved in the study were thoroughly informed about the research process and were instructed to abstain from eating until their blood was drawn for glucose testing [14].

Blood pressure measurements were recorded using a mercury sphygmomanometer calibrated for accuracy [15]. During the measurement, participants sat quietly for a minimum of 5 min, feet flat on the floor, arm supported at heart level [15]. Two separate measurements, taken at least 5 min apart, were averaged for the final blood pressure reading [15]. Modified Prasad’s classification was employed to assess the individual’s socioeconomic status [16], and household overcrowding was measured by the ratio of occupants to rooms [17].

Operational definitions were used for key measures in the study.

The waist–hip ratio (WHR) was computed by dividing the waist’s measurement, in centimetres, by the hip’s measurement, also in centimetres’. The hip measurement was taken at the most prominent portion of the buttocks, maintaining a parallel orientation to the floor [18]. To denote abdominal obesity, benchmarks of 0.95 for men and 0.80 for women were applied. [19]. Data were analysed using these average cut-off points [20].

Physical Activity: this was gauged based on World Health Organization (WHO) guidelines [21].

Body Weight: the weight of each participant was accurately recorded, barefoot and in lightweight attire, to the closest 100 g using a precision scale [22].

Height: participants’ height was determined while they stood erect, shoeless, back touching the wall, with their heels aligned and eyes focused straight ahead. The examiner noted the height on the wall, documenting it to the nearest 0.1 cm [23].

Body mass index (BMI): calculation for BMI was made using the equation weight (in kilograms) divided by the square of the height (in meters) [24]. Based on their BMI, participants were sorted into categories as per the WHO guidelines: underweight (BMI <18.5), normal weight (BMI = 18.5–24.9), overweight (BMI = 25.0–29.9), and obesity (BMI >30.0) [25].

Hypertension: the measurement of blood pressure was carried out with a properly adjusted mercury sphygmomanometer [26]. As per the 2018 ESC/ESH Guidelines for managing arterial hypertension, hypertension was characterised as having a blood pressure of 140/90 mm Hg or above [27].

Diabetes mellitus: The diagnosis of diabetes was made based on the International Diabetes Federation’s guidelines, which include fasting plasma glucose levels exceeding 7 mmol/l, a two-hour plasma glucose level above 11.1 mmol/l, HBA1C levels greater than 6.5%, and random plasma glucose levels surpassing 11.1 mmol/l alongside symptoms indicative of high blood sugar [28].

Overt hypothyroidism/subclinical hypothyroidism: the diagnosis of overt hypothyroidism was confirmed if TSH levels were higher than 4.50 µIU/mL, fT4 levels were less than 0.8 ng/dL, and fT3 levels were under 1.4 pg/mL. Subclinical hypothyroidism was identified if TSH levels exceeded 4.50 µIU/mL, fT4 levels were within the range of 0.8–1.8 ng/dL, and fT3 levels were between 1.4–4.4 pg/mL [29].

Hence, this study utilised a structured methodology and specific operational definitions to investigate the prevalence of several health conditions within the community [30].

### Statistical Analysis

Data were analysed utilising Microsoft Office Excel and SPSS Version 16.0. The study outlines counts and ratios pertaining to the variables under study. Descriptive statistical measures, including averages and standard deviations, were employed to showcase demographic and anthropometric attributes. Age stratification was performed during the statistical analysis, allowing for a more granular understanding of the prevalence and distribution of HTN and DM across different age groups. Furthermore, consideration was given to the treatment effect for HTN and DM due to varying treatment methods across the globe. The statistical analysis accounted for this treatment effect, allowing us to capture its potential impact on our study’s outcomes. Group statistics were clarified using frequencies and percentages, using the Chi-square test. Mean comparisons were carried out using the t-test. To evaluate the impact of factors such as age, gender, and obesity, binary logistic regression analysis was conducted. All statistical examinations were executed at a 5% significance threshold.

## 3. Results

This community-based cross-sectional study included 640 study subjects; of these, 120 had hypertension (HTN), and 200 had diabetes mellitus (DM). Table 1 shows that the majority of diseased patients belonged to the age group of more than 50 years, female category, lower socio-economic class, married group and those who had a family history. Almost every socio-demographic variable enlisted in Table 1 showed a statistically significant association between hypertension as well as diabetes mellitus.

Table 2 shows the distribution of behavioural aspects and eating habits of study subjects. Using tobacco products, consuming alcohol, type of diet as well as edible oil shows statistically significant association with diabetes mellites. Vigorous physical activity and walking, riding or cycling not show a statistically significant correlation with diabetes mellitus and hypertension.

Table 3 shows the distribution of anthropometric measurements such as BMI and W–H ratio of study subjects. Out of the total obese subjects, more than 62% had diabetes mellitus. Study subjects had a higher W–H ratio, in which approximately 40% had diabetes mellitus. Anthropometric variables in Table 3 showed statistically significant association between hypertension as well as diabetes mellitus.

**Figure 1:**
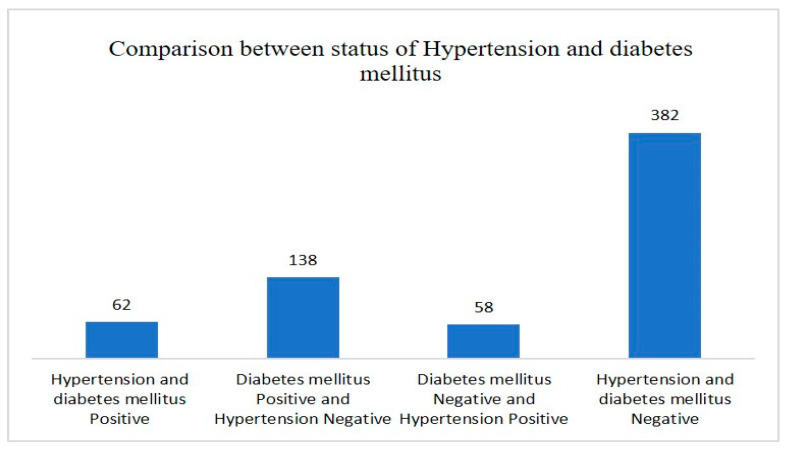
Comparison between the status of hypertension and diabetes mellitus of study subjects.

Figure 1 shows a comparison between the status of hypertension and diabetes mellitus of study subjects. Out of a total of 640 study subjects, 62 had both diseases, whereas 382 were disease-free subjects.

Table 4 displays the averages and fluctuations of thyroid function and serum glucose test results. It was noted that values of fT3, fT4, TSH, and glucose indicators such as FPG, PPG, and HbA1c exhibited congruence. Among patients with diabetes mellitus and hypertension, the thyroid function test’s variability was seen to be greatest for TSH levels and smallest for fT4 levels.

Table 5 illustrates 98 (25.7%) patients with overt hypothyroidism, 32 (8.4% patients with subclinical hypothyroidism and 252 (66.0%) were normal. In diabetes mellitus patients, 23 (19.2%) had overt, 7 (5.8%) had subclinical hypothyroidism, and 90 (75.0%) out of 120 diabetics were normal. Of hypertension patients, 60 (30.0%) had overt, 20 (10.0%) had subclinical hypothyroidism, and 120 (60.0%) were normal out of 200 hypertensives patients.

Table 6 depicts the binary logistic regression analyses and reports that married patients (OR = 2.26, 95% CI:1.62 to 3.15), patients belonging to lower and middle class (OR = 11.84, 95% CI: 4.60 to 30.48 and OR = 7.33, 95% CI: 2.58 to 20.77), using mustard oil (OR = 1.97, 95% CI: 1.04 to 3.73), consuming alcohol (OR = 4.14, 95%CI: 3.35 to 5.13), underweight (OR = 22.14, 95% CI: 1.35 to 363.63), and with higher W–H ratio (OR = 2.71, 95% CI: 1.73 to 4.24) were at higher risk for hypertension. Furthermore, patients having family history (OR= 3.76, 95% CI: 2.13 to 6.63), patients consuming alcohol (OR = 2.62, 95% CI: 2.16 to 3.16), and patients having higher W–H ratio (OR = 2.93, 95% CI: 2.05 to 4.20) were at higher risk for diabetes mellitus and patients having higher HbA1c (OR = 1.28, 95% CI: 0.76 to 2.23, OR = 2.66, 95% CI: 1.11 to 1.24) were at higher risk of hypertension and diabetes mellitus, respectively.

## 4. Discussion

Diabetes and hypertension are two significant non-communicable diseases often inadequately managed in Asia. Therefore, holistic and innovative methods that can enhance their detection, prevention, and control are essential. India dubbed the ‘Diabetes Capital of the World’, is home to 17% of the world’s diabetic population. The country currently hosts nearly 80 million people with diabetes, a number expected to rise to 135 million by 2045 [17]. As per the Fourth National Family Health Survey conducted in 2015–2016, adult hypertension prevalence was reported at 22.4% (men-13.6%, women-8.8%) [18].

Our community-based cross-sectional study examined 640 subjects, with 120 (18.75%) experiencing hypertension and 200 (31.25%) having diabetes mellitus. The lower prevalence of hypertension might be due to the study’s inclusion of adults aged 18 and above. Meanwhile, a study conducted by Kannan and Satyamoorthy, which focused on adults over 30 years, reported a 25.2% hypertension prevalence in rural areas [19].

Approximately 10% of the study participants had both diseases, while 60% were disease-free. Among all subjects with hypertension, 51.67% also had diabetes mellitus. This is similar to another study that reported hypertension in 54.1% of diabetic individuals [20]. In contrast, Khan et al. (2020) noted a hypertension prevalence of around 27.4%, a diabetes mellitus prevalence of 15.24%, and over 7% of subjects having both diseases [21]. Indeed, pharmacological treatments for diabetes, hypertension, and hypothyroidism are critical to managing these conditions.

For diabetes, treatment aims to maintain blood glucose levels within a normal range to prevent complications. Medications such as metformin, insulin, and sulfonylureas are commonly prescribed. Metformin works by decreasing glucose production by the liver and increasing insulin sensitivity, thereby improving glucose uptake by cells. Insulin therapy is critical for patients with type 1 diabetes and may also be required for patients with type 2 diabetes if other treatments are insufficient. Sulfonylureas stimulate the pancreas to produce more insulin. Other newer medication classes like GLP-1 agonists and SGLT2 inhibitors have additional benefits such as weight loss and cardiovascular risk reduction, respectively.

Hypertension treatment often involves lifestyle modifications, including dietary changes, increased physical activity, and weight loss. If these measures are not sufficient, medication may be necessary. Standard classes of antihypertensive drugs include diuretics, angiotensin-converting enzyme (ACE) inhibitors, angiotensin II receptor blockers (ARBs), calcium channel blockers, and beta-blockers. Each of these drug classes works in different ways to lower blood pressure, and they are often used in combination to achieve optimal control.

Hypothyroidism, an underactive thyroid, is typically treated with levothyroxine, a synthetic form of the hormone thyroxine (T4) that the thyroid gland naturally produces. The dosage of levothyroxine is carefully adjusted to restore thyroid hormone levels to a normal range, alleviating symptoms like fatigue, weight gain, and depression.

Intriguingly, there may be an overlap in the pharmacological management of these three conditions. Some studies have suggested that metformin may have a protective effect on the cardiovascular system, potentially benefiting patients with hypertension. Similarly, statins used in treating high cholesterol levels associated with diabetes and hypertension have shown some promise in improving thyroid function, although the research is less clear on this point.

The worldwide incidence of diabetes has seen a significant rise, escalating from 108 million in 1980 to 422 million in 2014. The diabetes rate in adults over the age of 18 increased from 4.7% in 1980 to 8.5% in 2014, with the brunt being borne by lower and middle-income nations [22]. Giri and colleagues’ research showed that 17.1% of the adult populace was diagnosed with hypertension, while 26% were identified with elevated blood pressure [20]. In alignment with our results, Subekti and team (2017) observed a notable linkage between diabetes, hypertension, and hypothyroidism [21].

In our research, a majority of the patients suffering from the conditions were over 50 years of age, female, from lower socio-economic groups, married, and those with a family history demonstrated a statistically significant correlation with hypertension and diabetes mellitus. This aligns with the findings of Mahmood and colleagues, who reported that the occurrence of hypertension was notably higher in individuals aged over 40 years [24]. Similarly, Vasan and the team’s research uncovered a significant link between hypertension and age [25].

Factors such as tobacco use, alcohol consumption, diet type, and type of cooking oil showed a statistically significant association with diabetes mellitus. Vigorous physical activity and walking or cycling did not show a statistically significant association with diabetes mellitus and hypertension.

Higher hypertension prevalence was observed in the upper class, and diabetes mellitus was more prevalent in the middle class, mirroring findings from a study conducted among Lucknow adults [26]. Societies undergoing economic and epidemiological transitions often record higher hypertension prevalence among upper socioeconomic groups [27].

Participants who were overweight or obese presented a higher incidence of hypertension in comparison to those of normal weight, whereas those underweight demonstrated a higher prevalence. A significant correlation was discovered between BMI and conditions like hypertension and diabetes mellitus. Moreover, over 62% of all obese individuals were diagnosed with diabetes mellitus. Subjects with a higher waist–hip ratio, around 40%, were found to have diabetes mellitus. Findings related to BMI echo those from studies performed in Odisha [28] and West Bengal [29]. Notable links were identified concerning the consumption of tobacco products, echoing earlier studies associating tobacco usage with hypertension [30,31]. Our research revealed a significant correlation between hypertension and smoking, a result also backed by a study undertaken in Maharashtra [32]. However, our study did not find a significant relationship between hypertension and alcohol consumption, contrary to other studies that recognise alcohol as an independent risk factor [33,34].

The National Urban Diabetes Survey (NUDS) conducted by Ramachandran A and colleagues (2001) [35], as well as Ravikumar P and team (2010) [36], documented a significant and independent relationship between diabetes and measures like BMI and waist–hip ratio (WHR). Pandya H and associates (2011) [37] discovered that obesity was more widespread among individuals with diabetes and that a larger waist circumference was frequently observed. Jayawardena R (2012) [38] deduced that a higher BMI and an elevated waist-–hip ratio heightened the risk of diabetes mellitus.

The co-occurrence of hypothyroidism, diabetes, and hypertension is a growing concern, and our study findings suggest an association that warrants further investigation. The prevalence of hypothyroidism among participants with diabetes and hypertension further highlights the interconnected nature of these conditions.

Our data showed that a significant percentage of subjects with hypertension also had diabetes mellitus, with a substantial overlap noted among individuals with hypothyroidism. This triangulation of diseases may suggest common underlying pathophysiological mechanisms or shared risk factors, such as age, socio-economic status, and certain lifestyle habits.

For hypertension, the odds ratio of 0.40 suggests that males have 40% lower odds of having hypertension compared to females. Regarding age, the odds ratio of 0.42 suggests that the younger age is associated with a decreased risk of hypertension. For diabetes mellitus, the odds ratios of 1.35 (gender) and 0.26 (age) suggest slightly increased odds of diabetes mellitus for males and decreased odds for individuals aged 50 or below, respectively.

Previous studies also found a connection between these conditions, supporting our findings. Furthermore, the demographic characteristics of patients diagnosed with these conditions in our study echo those found in another research. Notably, our research underscored that obesity, represented by BMI and waist–hip ratio, was significantly associated with diabetes and hypertension, a finding that is well corroborated in the literature.

Analysis involving multiple variables in our study showed that factors such as marital status, belonging to lower and middle classes, use of mustard oil, alcohol consumption, underweight status, and a higher W–H ratio posed a higher risk for hypertension. Furthermore, factors such as having a family history, alcohol consumption, and a higher W–H ratio increased the risk for diabetes mellitus.

Despite the wealth of information generated from this study about the relationship between hypothyroidism, hypertension, and diabetes mellitus, several limitations should be acknowledged. The cross-sectional nature of the study offers an overview of the condition’s prevalence at a particular point but does not support establishing temporal relationships or causality. Therefore, a longitudinal approach could offer a more solid basis for causal inferences.

Furthermore, the reliance on self-reported data for some risk factors and lifestyle variables might introduce recall bias or misreporting, potentially skewing the findings. Blood pressure, a key variable, was measured at a single point, failing to account for daily fluctuations and possibly offering a less reliable representation of the individual’s average levels.

The study’s exclusion criteria, omitting pregnant women, those under 18, individuals with cognitive impairments, and severely sick patients, limit the generalizability of the findings to these populations. The geographical focus of the study might also affect the extrapolation of the findings to other regions with differing demographic or socio-economic attributes.

Despite adjusting for multiple confounding factors, there could still be residual confounding due to unmeasured variables such as genetic predispositions or underlying health conditions. Lastly, while operational definitions were used for key measures in the study, they might not encompass the full range of conditions of interest, particularly for diseases like diabetes and hypothyroidism, which were diagnosed based on specific thresholds. Despite these limitations, the findings of this study contribute significantly to the understanding of hypothyroidism, hypertension, and diabetes mellitus and are valuable in guiding future research in this domain.

## 5. Conclusions

In our research, the relationship among hypertension, diabetes mellitus, and subclinical hypothyroidism took centre stage. The study brought to light that a majority, over fifty per cent, of subjects with hypertension also battled with diabetes mellitus. Distinct sociodemographic attributes, including those above the age of 50, females, individuals from lower socio-economic strata, the married population, and those with a familial history of such conditions surfaced as standalone risk factors for both hypertension and diabetes mellitus.

Additionally, the research underscored that lifestyle habits, such as tobacco usage, alcohol intake, and certain dietary preferences (inclusive of the type of cooking oil used), demonstrated a significant statistical association with diabetes mellitus. Consequently, based on these findings, we advocate for regular community-level screening programs for hypertension, diabetes mellitus, and subclinical hypothyroidism to facilitate early detection and management of these conditions.

Particularly concerning hypothyroidism, our study illuminated the necessity for consistent hypertension screening among subjects with subclinical hypothyroidism. The data exhibited that of the 120 hypertension-diagnosed individuals, 37.5% were also identified as diabetic, with the co-occurrence being markedly higher in individuals aged above 50 years, belonging to the lower socio-economic group, and those married.

Moreover, we observed a prevalent incidence of diabetes across various educational and occupational groups among hypertensive patients, with the highest frequency noted among postgraduates and professionals. These findings emphasise the need for a comprehensive approach to manage hypertension and diabetes, especially among the identified high-risk groups.

## Figures and Tables

**Table 1:** Distribution of socio-demographic parameters of study subjects.

Variables		HTN		*p* Value	DM		*p* Value
		Yes (*n* = 120)	No (*n* = 520)		Yes (*n* = 200)	No (*n* = 440)	
Age (in years)	≤ 50 (*n* = 359)	45	314	0.00	76	283	0.00
		12.5%	87.5%		21.2%	78.8%	
	>50 (*n* = 281)	75	206		124	157	
		26.7%	73.3%		44.1%	55.9%	
Gender	Male (*n* = 221)	35	186	0.17	96	125	0.00
		15.8%	84.2%		43.4%	56.6%	
	Female (*n* = 419)	85	334		104	315	
		20.3%	79.7%		24.8%	75.2%	
SES	Upper (*n* = 31)	14	17	0.00	7	24	0.18
		45.2%	54.8%		22.6%	77.4%	
	Middle (*n* = 199)	30	169		71	128	
		15.1%	84.9%		35.7%	64.3%	
	Lower (*n* = 410)	76	334		122	288	
		18.5%	81.5%		29.8%	70.2%	
Marital status	Married (*n* = 477)	70	407	0.00	116	361	0.00
		14.7%	85.3%		24.3%	75.7%	
	Unmarried (*n* = 163)	50	113		84	79	
		30.7%	69.3%		51.5%	48.5%	
Education	Postgraduate (*n* = 32)	12	20	0.00	12	20	0.00
		37.5%	62.5%		37.5%	62.5%	
	Graduate (*n* = 96)	27	69		26	70	
		28.1%	71.9%		27.1%	72.9%	
	Intermediate (*n* = 90)	7	83		15	75	
		7.8%	92.2%		16.7%	83.3%	
	High school (*n* = 46)	0	46		31	15	
		0.0%	100.0%		67.4%	32.6%	
	Middle school (*n* = 95)	16	79		32	63	
		16.8%	83.2%		33.7%	66.3%	
	Primary school (*n* = 47)	11	36		23	24	
		23.4%	76.6%		48.9%	51.1%	
	Illiterate (*n* = 234)	47	187		61	173	
		20.1%	79.9%		26.1%	73.9%	
Occupation	Professional (*n* = 50)	12	38	0.00	18	32	0.00
		24.0%	76.0%		36.0%	64.0%	
	Shop owner (*n* = 110)	28	82		63	47	
		25.5%	74.5%		57.3%	42.7%	
	Daily wager (*n* = 107)	0	107		26	81	
		0.0%	100.0%		24.3%	75.7%	
	Unemployment (*n* = 18)	0	18		6	12	
		0.0%	100.0%		33.3%	66.7%	
	Housewife (*n* = 355)	80	275		87	268	
		22.5%	77.5%		24.5%	75.5%	
Type of family	Nuclear (*n* = 401)	60	341	0.00	86	315	0.00
		15.0%	85.0%		21.4%	78.6%	
	Joint (*n* = 239)	60	179		114	125	
		25.1%	74.9%		47.7%	52.3%	
Family income	< 10,000 (*n* = 406)	60	346	0.00	106	300	0.00
		14.8%	85.2%		26.1%	73.9%	
	≥ 10,000 (*n* = 234)	60	174		94	140	
		25.6%	74.4%		40.2%	59.8%	
Family history	Yes (*n* = 477)	88	389	0.73	124	353	0.00
		18.4%	81.6%		26.0%	74.0%	
	No (*n* = 163)	32	131		76	87	
		19.6%	80.4%		46.6%	53.4%	

**Table 2:** Distribution of behaviour and food practices of study subjects.

		HTN		*p* Value	DM		*p* Value
		Yes (*n* = 120)	No (*n* = 520)		Yes (*n* = 200)	No (*n* = 440)	
Type of diet	Vegetarian (*n* = 273)	59	214	0.11	98	175	0.02
		21.6%	78.4%		35.9%	64.1%	
	Non-Vegetarian (*n* = 367)	61	306		102	265	
		16.6%	83.4%		27.8%	72.2%	
Type of oil	Refined Oil (*n* = 332)	78	254	0.00	102	230	0.01
		23.5%	76.5%		30.7%	69.3%	
	Mustard Oil (*n* = 136)	18	118		55	81	
		13.2%	86.8%		40.4%	59.6%	
	Other (*n* = 172)	24	148		43	129	
		14.0%	86.0%		25.0%	75.0%	
Do you ever use tobacco	Yes (*n* = 159)	28	131	0.67	59	100	0.06
		17.6%	82.4%		37.1%	62.9%	
	No (*n* = 481)	92	389		141	340	
		19.1%	80.9%		29.3%	70.7%	
Do you ever use alcohol	Yes (*n* = 105)	16	89	0.31	52	53	0.00
		15.2%	84.8%		49.5%	50.5%	
	No (*n* = 535)	104	431		148	387	
		19.4%	80.6%		27.7%	72.3%	
Do you have symptoms of diabetes	Yes (*n* = 281)	81	200	0.00	160	121	0.00
		28.8%	71.2%		56.9%	43.1%	
	No (*n* = 359)	39	320		40	319	
		10.9%	89.1%		11.1%	88.9%	
Over Crowding	Present (*n* = 229)	45	184	0.66	62	167	0.08
		19.7%	80.3%		27.1%	72.9%	
	Absent (*n* = 411)	75	336		138	273	
		18.2%	81.8%		33.6%	66.4%	
Vigorous Physical Activity	Yes (*n* = 170)	30	140	0.66	47	123	0.23
		17.6%	82.4%		27.6%	72.4%	
	No (*n* = 470)	90	380		153	317	
		19.1%	80.9%		32.6%	67.4%	
Walk or bicycle for at least 10 min	Yes (*n* = 173)	29	144	0.43	49	124	0.33
		16.8%	83.2%		28.3%	71.7%	
	No (*n* = 467)	91	376		151	316	
		19.5%	80.5%		32.3%	67.7%	

**Table 3:** Distribution of anthropometric measurements of study subjects.

		Total	HTN		*p* Value	DM		*p* Value
			Yes (*n* = 120)	No (*n* = 520)		Yes (*n* = 200)	No (*n* = 440)	
BMI	<18.5 (Underweight) (*n* = 44)	44	0	44	0.00	8	36	0.00
		100.0%	0.0%	100.0%		18.2%	81.8%	
	18.5–24.9 (Normal) (*n* = 378)	378	75	303		108	270	
		100.0%	19.8%	80.2%		28.6%	71.4%	
	25–29.9 (Overweight) (*n* = 173)	173	28	145		56	117	
		100.0%	16.2%	83.8%		32.4%	67.6%	
	>30 (Obesity) (*n* = 45)	45	17	28		28	17	
		100.0%	37.8%	62.2%		62.2%	37.8%	
W–H ratio	≤0.875 (*n* = 277)	277	30	247	0.00	55	222	0.00
		100.0%	10.8%	89.2%		19.9%	80.1%	
	> 0.876 (*n* = 363)	363	90	273		145	218	
		100.0%	24.8%	75.2%		39.9%	60.1%	

**Table 4:** Mean and standard deviation of thyroid function and glycemic indicators test.

	fT3 (pg/mL)	fT4 (ng/dL)	TSH (μiU/mL)	FPG (mg/dL)	PPG (mg/dL)	HbA1c (%)
DM	3.4 ± 9.8	0.9 ± 0.7	3.3 ± 6.2	153.1 ± 68.0	213.2 ± 97.2	8.3 ± 3.2
HTN	3.1 ± 9.9	1.1 ± 0.8	3.8 ± 10.4	92.7 ± 10.9	113.3 ± 21.8	6.1 ± 0.6
DM + HTN	3.4 ± 9.2	1.2 ± 0.7	3.1 ± 4.8	128.8 ± 45.2	191.0 ± 67.8	7.9 ± 1.8

**Table 5:** Relationship between hypothyroidism with hypertension in diabetes mellitus patients.

	Hypothyroidism		Normal *n* (%)
	Overt, *n* (%)	Subclinical, *n*(%)	
DM (*n* = 120)	23 (19.2%)	7 (5.8%)	90 (75.0%)
HTN (200)	60 (30.0%)	20 (10.0%)	120(60.0%)
DM + HTN (*n* = 62)	15 (24.2%)	5 (8.1%)	42(67.7%)
Total (*n* = 382)	98(25.7%)	32(8.4%)	252(66.0%)

**Table 6:** Binary logistic regression analysis.

	HTN				DM			
	*p* Value	Odds Ratio	95% C.I. for Odds Ratio		*p* Value	Odds Ratio	95% C.I. for Odds Ratio	
			Lower	Upper			Lower	Upper
Gender(male)	0.02	0.40	0.19	0.85	0.42	1.35	0.66	2.79
Age (≤50 years)	0.00	0.42	0.23	0.75	0.00	0.26	0.13	0.49
SES (middle)	0.00	11.84	4.60	30.48	0.02	0.31	0.11	0.85
SES (lower)	0.00	7.33	2.58	20.77	0.25	0.53	0.18	1.55
Family Income (<10,000)	0.48	0.75	0.34	1.65	0.17	1.84	0.77	4.40
Type of family (Nuclear)	0.13	0.59	0.30	1.17	0.00	0.20	0.10	0.41
Marital Status (married)	0.00	2.26	1.62	3.15	0.70	0.94	0.69	1.28
Type of Diet (Vegetarian)	0.24	0.73	0.43	1.24	0.03	0.51	0.28	0.93
Type of oil (Mustard Oil)	0.04	1.97	1.04	3.73	0.65	1.17	0.60	2.29
Type of oil (Other)	0.03	2.40	1.09	5.26	0.76	0.90	0.45	1.80
Do you ever use tobacco (Yes)	0.45	1.33	0.64	2.74	0.80	1.11	0.50	2.46
Do you ever use alcohol (Yes)	0.00	4.14	3.35	5.13	0.00	2.62	2.16	3.16
Family history (Yes)	0.57	1.19	0.66	2.12	0.00	3.76	2.13	6.63
Do you have symptoms of diabetes (yes)	0.00	0.42	0.24	0.74	0.00	0.11	0.06	0.19
BMI (<18.5 Underweight)	0.03	22.14	1.35	363.63	0.15	1.80	0.81	4.00
BMI (Overweight)	0.00	0.04	0.02	0.06	0.37	0.84	0.57	1.23
BMI (Obesity)	0.00	0.09	0.05	0.17	0.00	0.24	0.13	0.46
W–H ratio (>0.875)	0.00	2.71	1.73	4.24	0.00	2.93	2.05	4.20
fT3 (pg/mL)	0.49	0.65	0.30	1.45	0.26	0.94	0.66	3.40
fT4 (ng/dL)	0.22	0.68	0.28	1.22	0.04	0.30	0.12	0.34
TSH (μiU/mL)	0.00	1.21	1.10	2.15	0.67	0.83	0.58	1.17
FPG (mg/dL)	0.21	0.84	0.54	1.35	0.02	0.62	0.39	1.04
PPG (mg/dL)	0.03	1.86	1.14	3.64	0.87	1.06	0.51	2.16
HbA1c (%)	0.01	1.28	0.76	2.23	0.00	2.66	1.11	1.24

## Data Availability

The datasets analysed in the current study are available from the corresponding author on reasonable request.
